# The impact of postmastectomy radiotherapy on cT1-2N1 breast cancer patients with ypN0 after neoadjuvant chemotherapy: a retrospective study based on real-world data

**DOI:** 10.1007/s12672-022-00609-8

**Published:** 2023-01-07

**Authors:** Yuran Dai, Shishi Ma, Ailin Lan, Yihua Wang, Yu Wang, Yudi Jin, Nan Ding, Linshan Jiang, Zhenrong Tang, Xuedong Yin, Yang Peng, Shengchun Liu

**Affiliations:** 1 Department of Breast and Thyroid Surgery, The First Affiliated Hospital of Chongqing Medical University, Chongqing, China; 2 Department of Oncology, The First Affiliated Hospital of Chongqing Medical University, Chongqing, China; 3 Department of General Surgery, State Key Laboratory of Complex Severe and Rare Diseases, Peking Union Medical College Hospital, Chinese Academy of Medical Sciences and Peking Union Medical College, Beijing, People’s Republic of China; 4 Department of Pathology, Chongqing University Cancer Hospital, Chongqing, China

**Keywords:** Breast cancer, Neoadjuvant chemotherapy, Post mastectomy radiotherapy

## Abstract

**Background:**

The role of postmastectomy radiation therapy (PMRT) in clinical T1-2N1 breast cancer patients who achieve axillary pathological complete response (ypN0) after neoadjuvant chemotherapy (NAC) is controversial.

**Methods:**

Data from cT1-2N1 breast cancer patients who converted to ypN0 after NAC and subsequent surgery were retrospectively analyzed. Disease-free survival (DFS) and overall survival (OS) were estimated using the Kaplan‒Meier method. Univariate and multivariate Cox regression models were applied to investigate the correlations between clinical or pathological parameters and survival.

**Results:**

From 2012–2019, we identified 116 cases for analysis, including 31 (26.7%) who received PMRT and 85 (73.3%) who did not. At a median follow-up time of 56.4 months, the 5-year DFS and OS rates were 90.2% and 96.7% with PMRT and 93.7% and 97.3% without PMRT, respectively. PMRT did not affect either DFS (p = 0.234) or OS (p = 0.878). On multivariate analyses, no differences in DFS or OS between the two groups were detected, taking into consideration the following factors: age, molecular subtype, Ki67 index, cT stage, and in-breast pathologic complete response (DFS: HR 2.260; 95% CI 0.465–10.982; p = 0.312. OS: HR 1.400; 95% CI 0.138–14.202; p = 0.776). This nonsignificant difference was also consistent in subgroup analyses (all p > 0.05).

**Conclusions:**

PMRT has limited ability to confer DFS or OS benefits for cT1-2N1 breast cancer patients who achieved axillary pathological complete response after NAC and total mastectomy. It is imperative to conduct prospective studies to investigate the safety and feasibility of omitting PMRT.

*Trial registration:* This research was approved by the Ethics Committee of The First Affiliated Hospital of Chongqing Medical University (ID: No. 2021–442).

## Background

Comprehensive management of breast cancer mainly includes surgery, chemotherapy, radiotherapy, and endocrine therapy. Numerous randomized clinical trials and meta-analyses confirmed the satisfying local control and long-term survival brought by postmastectomy radiotherapy (PMRT) [1–5]. According to the National Comprehensive Cancer Network (NCCN), the initial status of the primary breast tumor and regional lymph node assist in making a preliminary determination on the delivery of PMRT.

Neoadjuvant chemotherapy (NAC), a recognized option for locally advanced breast cancer, has gradually played a role in operable early-stage patients [6, 7]. In recent years, data from several studies have suggested that NAC can significantly decrease the size of tumor lesions and enlarged axillary lymph nodes [8], and even achieve pathological downstaging in 40% of patients [9–11].

Apparently, it is difficult to accurately assess the prognosis in NAC patients since the postoperative pathological status of the lymph node differs from that before initial treatment [12, 13]. Under this circumstance, the decision of PMRT for NAC patients becomes complex and tricky, especially for those without residual nodal disease pathologically (ypN0) after NAC. This cohort accounted for 20–40% of all NAC patients [7].

In this study, we investigated the impact of PMRT for cT1-2N1 breast cancer patients with ypN0 after NAC to explore whether this cohort can safely omit delivery of PMRT.

## Methods

### Patients

**Fig. 1:**
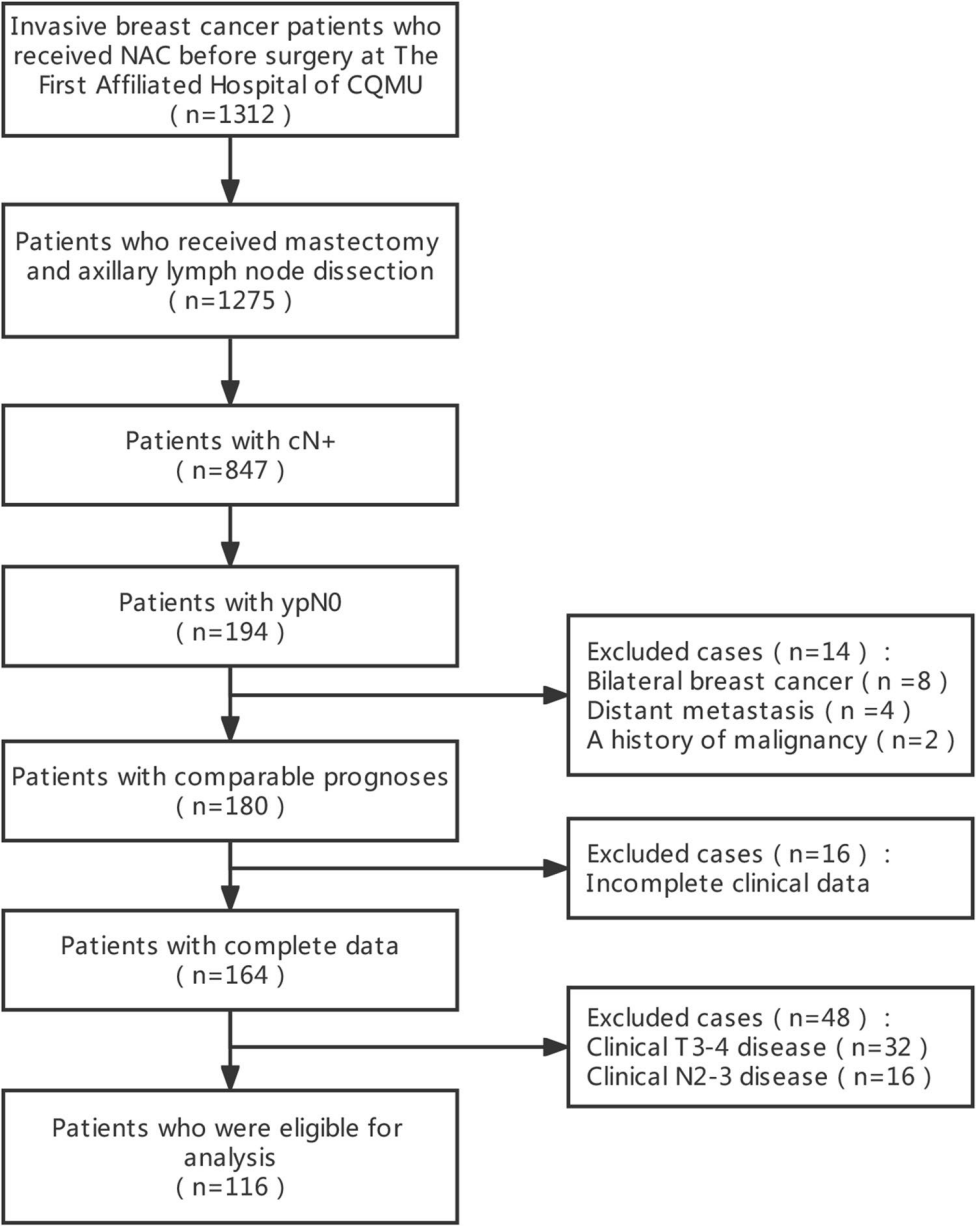
Flow chart of the patient selection

There were 1312 patients with pathologically diagnosed primary breast carcinoma who received NAC at the First Affiliated Hospital of Chongqing Medical University from January 2012 to December 2019. The criteria for selecting the participants were as follows: (a) patients whose diagnoses were confirmed by histology accepted  ≥ 2 cycles of NAC and subsequent mastectomy and complete axillary lymph node dissection; (b) patients with clinical N + disease before initial treatment; and (c) patients achieved ypN0 after NAC and mastectomy. Individuals were excluded from the study on the basis of the following criteria: (a) synchronous bilateral breast cancer; (b) distant metastasis before NAC; (c) another previous or concurrent malignancy diagnosis; (d) incomplete clinical data; (e) clinical T3-4 disease before NAC; and (f) clinical N2-3 disease before NAC. Ultimately, 116 patients were eligible for analysis (Fig. 1). We collected the details related to the demographic and pathological characteristics. All tumor specimens were reviewed by two experienced pathologists. The status of ER, PR, HER2, and the Ki67 index were measured by immunohistochemistry (IHC). We applied imaging studies to identify the clinical stage based on the 8th edition of the American Joint Committee on Cancer TNM Staging System. Patients with cT3-4 or cN2-3 disease, namely locally advanced breast cancer patients, were excluded because they are standard indications for PMRT regardless of treatment response. Patient data acquisition, analysis, and reporting were approved by our institutional review board.

### Treatment

Due to the considerable controversy over the effect of PMRT, there are no authorized recommendations with respect to PMRT delivery in cT1-2N1 patients with ypN0 after NAC. Under this circumstance, the radiation oncology department offered each patient an individual consultation. Together, the patient and her physicians made the final decision. For the standard PMRT protocol, the axillary level III, the supraclavicular fossa, and ± mammaria interna were irradiated with 50 Gy in 25 fractions over 5 weeks, with or without a boost. The chest wall was not routinely irradiated. Radiation to the mammaria interna depended on whether the tumor was located in the inner or central quadrant. All patients received other systemic treatments following nationally accepted guidelines or consensus.

### Follow-up

Setting the cutoff date to November 1, 2021, patients returned for regular assessment following a proposed schedule. Within 2 years after surgery, the follow-up was arranged to be once every 3 months; 3–5 years, once every 6 months; and more than 5 years, once a year. The specific frequencies and examinations required vary considering the actual situation of patients. Therefore, a track record for any event such as recurrence, metastasis, or death was established. Overall survival (OS) was calculated from the surgery date to the date of death from any cause or to the last visit. Disease-free survival (DFS) was defined as the length of time from surgery to local, regional, distant recurrence, second primary cancer, death from any cause, or the last visit.

### Statistical analysis

The curves for OS and DFS were constructed using the Kaplan‒Meier method and compared using the log-rank test. The characteristics of the PMRT and no-PMRT groups were compared using the χ2 test or Fisher’s exact test. The targeted and endocrine therapy analyses were carried out in corresponding patients, that is, in HER2 + and HR + cohorts, respectively. By the Cox regression model, we analyzed the impact of the following factors on survival: delivery of PMRT (no or yes); age (< 50 or ≥ 50 years); molecular subtype (HR + HER2− or HR + HER2 + or HR−HER2− or HR−HER2 +); Ki67 index (≤ 30 or > 30); cT (T1 or T2) stage at diagnosis; and in-breast pathologic complete response (nonpCR or pCR). Subgroup analyses were carried out to distinguish appropriate patients to omit PMRT. Hazard ratios (HRs) and 95% confidence intervals (CIs) were applied. Statistical significance was defined as a two-sided p < 0.05. Data management and analysis were performed using SPSS software (version 26).

## Results

### Demographic, clinicopathological, and treatment characteristics

Of 1312 pathologically invasive breast cancer patients who received NAC in our institution, 116 were eligible for this study (Fig. 1). The baseline characteristics are displayed in Table 1. In total, 31 patients (26.7%) received PMRT and 85 patients (73.3%) did not. The mean age at diagnosis was 60.5 years (range, 21–67). Most patients had cT2 (84.5%) disease. Only 1 patient showed lymph vascular invasion. In the immunohistochemical analysis, more than half of the patients were HR-positive (56.9%) and HER2-positive (58.6%). Regarding the in-breast response, most patients (86.2%) did not achieve pCR after NAC. A medium of 16 (range, 6–32) lymph nodes were resected. Notably, we analyzed the administration of endocrine and targeted therapy in populations with corresponding indications, which are HR-positive and HER2-positive patients, respectively. In contrast with the patients omitting PMRT, those who received PMRT had a stronger inclination to receive another systemic therapy in combination (endocrine therapy: 52.1% vs. 83.3%, p = 0.025; targeted therapy: 15.7 vs. 70.6%, p < 0.001). No statistically significant differences between the PMRT and no-PMRT groups were evident concerning the age at diagnosis, immunohistochemical terms, clinical T stage, or in-breast response. All patients received NAC with a median of 4 cycles (mean, 4.03; range: 3–6). A combination of anthracycline and taxane was chosen the most (90.5%), while less common regimens were anthracycline-based (7.8%) and taxane-based (1.7%) chemotherapy.

**Table 1:** Baseline characteristics of all patient

Characteristics	No PMRT		PMRT		Total		p value
	No	%	No	%	No	%	
Age							0.475
< 50	42	49.4	13	47.4	55	47.4	
≥ 50	43	50.6	18	52.6	61	52.6	
Hormonal receptor status							0.878
Negative	37	43.5	13	41.9	50	43.1	
Positive	48	56.5	18	58.1	66	56.9	
HER-2 receptor status							0.617
Negative	34	40.0	14	45.2	48	41.4	
Positive	51	60.0	17	54.8	68	58.6	
Molecular subtype							0.916
HR + HER2−	21	24.7	8	25.8	29	25.0	
HR + HER2 +	27	31.8	10	32.3	37	31.9	
HR−HER2−	13	15.3	6	19.4	19	16.4	
HR−HER2 +	24	28.2	7	22.6	31	26.7	
Ki67							0.831
≤ 30	53	62.4	20	64.5	73	62.9	
> 30	32	37.6	11	35.5	43	37.1	
Clinical T stage							0.777
T1	14	16.5	4	12.9	18	15.5	
T2	71	83.5	27	87.1	98	84.5	
In-breast response							0.762
Non-pCR	74	87.1	26	83.9	100	86.2	
pCR	11	12.9	5	16.1	16	13.8	
Endocrine therapy							0.025
No	23	47.9	3	16.7	26	39.4	
Yes	25	52.1	15	83.3	40	60.6	
Targeted therapy							< 0.001
No	43	84.3	5	29.4	48	70.6	
Yes	8	15.7	12	70.6	20	29.4	

*HR* Hormone receptor, *HER2* Human epidermal growth factor receptor 2, *pCR* Pathological complete response

### Disease-free and overall survival

Overall, the median follow-up time was 56.4 months (range, 7–114 months) with 43.7 and 68.6 months in the PMRT and no-PMRT group, respectively. Five patients (4.3%) were lost to follow-up. Until the deadline of the present study, 4 patients (3.4%) had died, 1 of whom was in the PMRT group while 3 were in the no-PMRT group. There was no locoregional recurrence. Seven patients (6.0%) experienced distant metastases that were first found in the lung, liver, brain, bone, or contralateral breast. Among them, 3 were in the PMRT group and 4 were in the no-PMRT group.

**Fig. 2:**
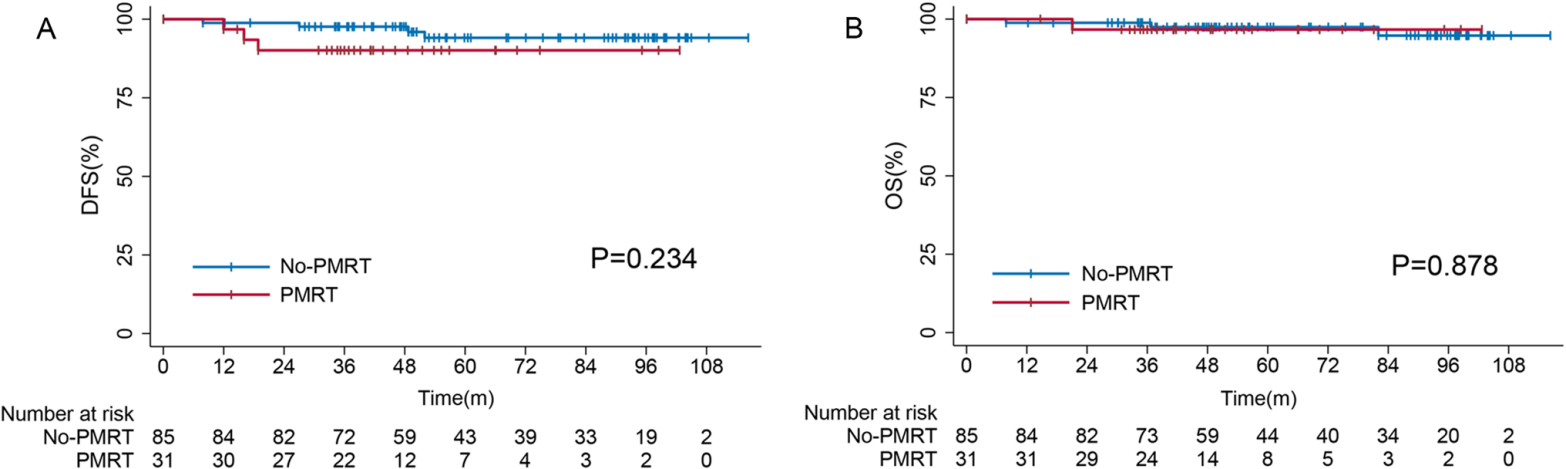
Kaplan–Meier survival curve according to delivery of PMRT. **A** Disease-Free survival. **B** Overall survival. Legends: **A** No significant differences in DFS were shown between the PMRT group and no-PMRT group (5-year DFS: 90.2% vs 93.7%, p = 0.234). **B** No significant differences in OS were shown between the PMRT group and no-PMRT group (5-year OS: 96.7% vs 97.3%, p = 0.878)

As illustrated in Fig. 2, there was no significant difference in DFS (p = 0.234) or OS (p = 0.878) for all included patients. The 5-year DFS and OS rates were 92.3% (95% CI 86.6–98.0%) and 97.0% (95% CI 93.7–99.2%), respectively. Patients with PMRT showed a 5-year DFS rate of 90.2% (95% CI 79.7–99.7%), while those without PMRT had a rate of 93.7% (95% CI 87.6–99.8%). Correspondingly, the 5-year OS rates were 96.7% (95% CI 90.4–99.4%) and 97.3% (95% CI 93.5–99.7%).

**Fig. 3:**
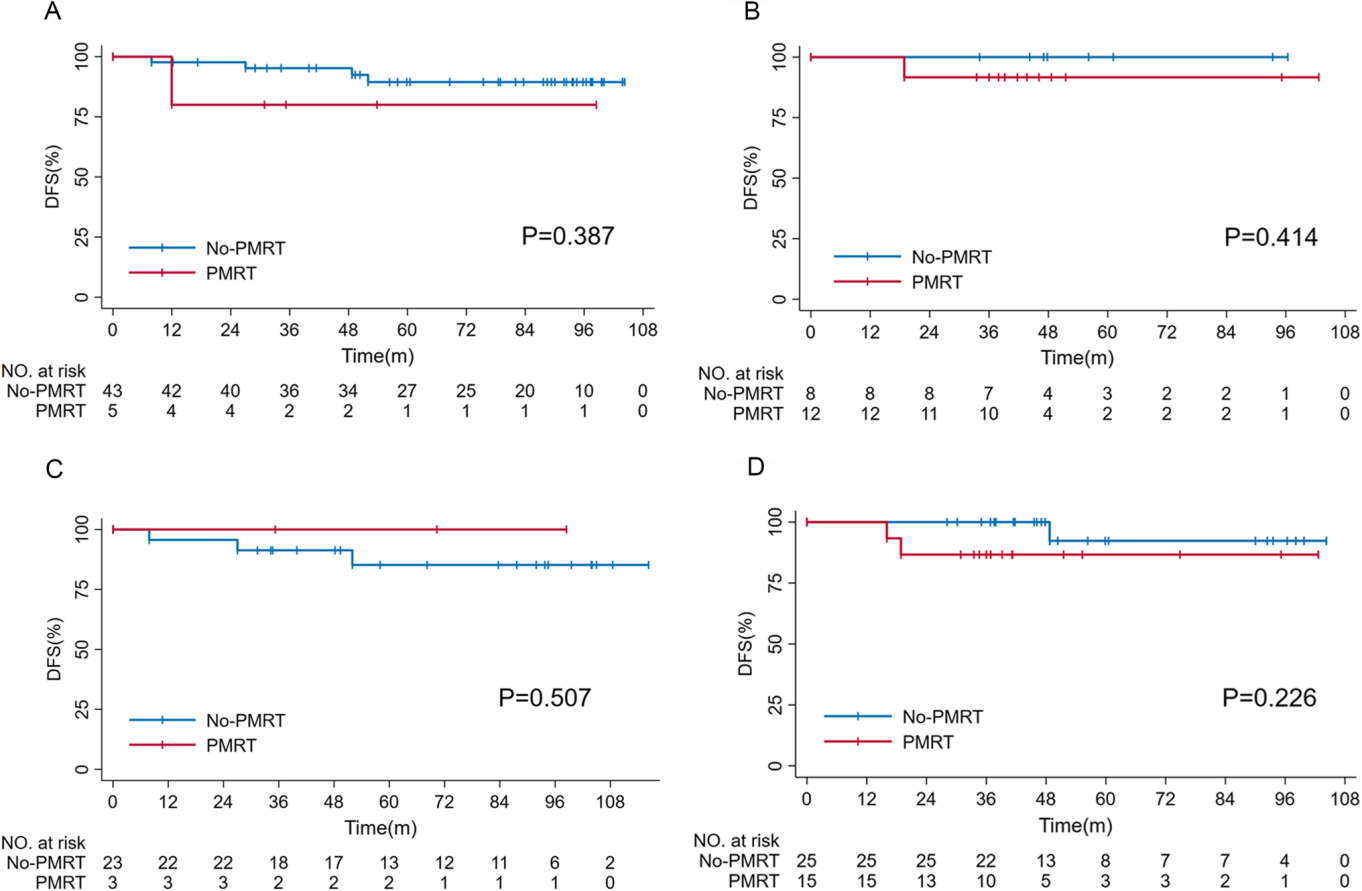
Kaplan–Meier survival curve for HR( +) and HER2( +) patients with different systemic treatment protocols. **A** DFS in HR( +) patients without endocrine therapy. **B** DFS in HR( +) patients with endocrine therapy. **C** DFS in HER2( +) patients without targeted therapy. **D** DFS in HER2( +) patients with targeted therapy. Legends: No significant differences in DFS were shown according to delivery of PMRT (all p > 0.05)

Furthermore, due to the inclination in the administration of endocrine and targeted therapy at baseline, we conducted disease-free survival analyses in HR-positive or HER2-positive cohorts. As shown in Fig. 3, radiation does not improve DFS in HR( +) patients with or without endocrine treatment (all p > 0.05). Similarly, radiation does not bring significant benefit to DFS in HER2( +) patients, regardless of the situation of targeted treatment (all p > 0.05).

### Univariate and multivariate analyses

The correlations between the parameters and survival are displayed in Tables 2 and 3. On univariate analysis, there was no evidence that any clinicopathological factors had an influence on DFS or OS (all p > 0.05), including PMRT (DFS: HR 2.437; 95% CI 0.537–11.062; p = 0.248 vs. OS: HR 1.198; 95% CI 0.118–12.157; p = 0.879). The same situation occurred in the multivariate analysis. No association was observed between PMRT and DFS (HR 2.260; 95% CI 0.465–10.982; p = 0.312) or OS (HR 1.400; 95% CI 0.138–14.202; p = 0.776).

**Table 2:** Univariate and multivariate analyses of disease-free survival for all included patients

Characteristics	Univariate		Multivariate	
	HR (95%CI)	p value	HR (95%CI)	p value
Radiotherapy				
No PMRT	Reference	–	Reference	–
PMRT	2.437(0.537–11.062)	0.248	2.260(0.465–10.982)	0.312
Age				
< 50	Reference	–	Reference	–
≥ 50	1.234(0.276–5.516)	0.783	1.376(0.300–6.299)	0.681
Hormonal receptor status				
Negative	Reference	–	–	–
Positive	4.890(0.588–40.684)	0.142	–	–
HER-2 receptor status				
Negative	Reference	–	–	–
Positive	4.297(0.517–35.748)	0.177	–	–
Molecular subtype				
HR + HER2−	Reference	–	Reference	–
HR + HER2 +	3.940(0.459–33.836)	0.211	4.181(0.432–40.480)	0.217
HR−HER2−	–	0.979	–	0.979
HR−HER2 +	0.886(0.055–14.226)	0.932	0.834(0.048–14.529)	0.901
Ki67				
≤ 30	Reference	–	Reference	–
> 30	0.767(0.149–3.954)	0.751	1.828(0.297–11.255)	0.515
Clinical T stage				
T1	Reference	–	Reference	–
T2	1.088(0.131–9.039)	0.938	1.243(0.144–10.711)	0.843
In-breast response				
Non-pCR	Reference	–	Reference	–
pCR	2.433(0.472–12.554)	0.288	2.388(0.410–13.923)	0.333
Endocrine therapy				
No	Reference	–	–	–
Yes	0.706(0.140–3.569)	0.674	–	–
Targeted therapy				
No	Reference	–	–	–
Yes	0.541(0.062–4.733)	0.579	–	–

*HR* Hormone receptor, *HER2* Human epidermal growth factor receptor 2, *pCR* Pathological complete response

**Table 3:** Univariate and multivariate analyses of overall survival for all included patients

Characteristics	Univariate		Multivariate	
	HR (95%CI)	p value	HR (95%CI)	p value
Radiotherapy				
No PMRT	Reference	–	Reference	–
PMRT	1.198(0.118–12.157)	0.879	1.400(0.138–14.202)	0.776
Age				
< 50	Reference	–	Reference	–
≥ 50	1.008(0.141–7.193)	0.994	1.444(0.184–11.354)	0.727
Hormonal receptor status				
Negative	Reference	–	–	–
Positive	0.747(0.105–5.303)	0.770	–	–
HER-2 receptor status				
Negative	Reference	–	–	–
Positive	46.469(0.011–201,331.429)	0.369	–	–
Molecular subtype				
HR + HER2−	Reference	–	Reference	–
HR + HER2 +	48740.179(0–6.860E155)	0.952	72158.703(0–1.532E162)	0.952
HR−HER2−	0.976(0–1.390E250)	1.000	1.149(0–9.609E304)	1.000
HR−HER2 +	55792.973(0–7.852E155)	0.951	76858.920(0–1.630E162)	0.951
Ki67				
≤ 30	Reference	–	Reference	–
> 30	0.682(0.071–6.58)	0.741	1.349(0.111–16.453)	0.815
Clinical T stage				
T1	Reference	–	Reference	–
T2	0.201(0.028–1.435)	0.110	0.239(0.029–1.983)	0.185
In-breast response				
Non-pCR	Reference	–	Reference	–
pCR	0.039(0–8258.959)	0.605	0(0–5.958E190)	0.964
Endocrine therapy				
No	Reference	–	–	–
Yes	0.008(0–1517.975)	0.435	–	–
Targeted therapy				
No	Reference	–	–	–
Yes	0.029(0–348.247)	0.459	–	–

*HR* Hormone receptor, *HER2* Human epidermal growth factor receptor 2, *pCR* Pathological complete response

### Subgroup analysis

Subgroup analyses (Fig. 3) revealed comparable DFS and OS between patients with or without PMRT across different subgroups, including patients older than 50 years or younger; negative or positive hormonal receptor status; negative or positive HER-2 receptor status; Ki67 index higher than 30 or lower; clinical T1 or T2 stage; and achievement of in-breast pCR or not (all p > 0.05).

## Discussion

Traditionally, the indications for PMRT largely depend on the initial status of the tumor. With the increasing application of NAC in early-stage disease, confusion and controversy for subsequent locoregional management have emerged [12, 13]. Approximately 20–40% of patients have lesions that shrink in the primary tumor or involve axillary nodes [7]. Therefore, the standard indications need to be updated urgently.

Due to the lack of data on prospective phase III trials, convincing criteria to select patients who would benefit from PMRT remain unclear. As stated by the current NCCN guidelines, the determination of PMRT for patients after NAC should consider the highest stage, either clinical or pathological. Notably, the effect of NAC on the initial stage was not reflected in the guidelines [2, 14]. The American Society of Clinical Oncology (ASCO) and American Society for Radiation Oncology (ASTRO) recommend PMRT for clinical stage II patients with positive axillary lymph nodes after NAC. However, there is still no clear recommendation for cT1-2N1 patients who converted to ypN0 [15].

The results from prospective trials are needed to firmly establish an appropriate system for locoregional treatment decisions. The RAPCHEM BOOG 2010–03 trial is designed to clarify the role of PMRT in cT1–2N1 patients according to ypN status [16]. In the low-risk group, which consisted of ypN0 patients, radiotherapy did not result in significantly altered 5-year LRR. The ongoing NSABP51-B51/RTOG-1304 trial (NCT01872975) aims to directly compare the prognosis of early-stage ypN0 patients with or without PMRT. The ATNEC trial (NCT04109079) investigated axillary management including axillary lymph node dissection and radiotherapy, in T1-3N1 patients without nodal metastases post NAC.

Previous retrospective studies have drawn conflicting results on the impact of PMRT on survival in ypN0 patients after NAC. To date, two large-sample-size studies are derived from the National Cancer Database (NCDB) [17, 18]. Rusthoven et al. analyzed 3040 ypN0 patients who underwent NAC and modified radical mastectomy. Various analyses consistently showed that PMRT significantly reduced the risk of death, indicating that these patients could benefit from PMRT in terms of OS [17]. However, according to Kantor et al., PMRT did not improve OS in the ypN0 cohort, regardless of their clinicopathological features. This is consistent in most high-risk subgroups, except for patients with ER/PR-negative disease [18]. Unfortunately, information on recurrence is unavailable in the NCDB, so OS was the only long-term outcome studied. In addition, insufficient data on HER-2 and targeted therapy hindered investigations into the role of PMRT in real-world conditions.

In the greater part of the literature, which has been small-sample-size and retrospective, controversy remains. MD Anderson reported a survival benefit of PMRT in 106 patients achieving a pCR after NAC [19]. It was associated with an improvement in 10-year local–regional recurrence (LRR), distant metastasis-free survival (DMFS), and OS for patients with stage III disease (LRR: 7.3% in the irradiated group vs. 33% in the nonirradiated group, p = 0.040) (DMFS: 87.9% vs. 40.7%, p = 0.0006) (OS: 77.3% vs. 33.3%, p = 0. 0016). In contrast to the MDACC findings, small institutional series in France and Korea did not detect any evidence for the benefit of PMRT for ypN0 patients [20, 21]. Le Scodan et al. analyzed 134 ypN0 patients, concluding that receiving PMRT or not had nothing to do with the risk of distant metastasis, locoregional recurrence, or death (p > 0.1) [20]. Similarly, Shim et al. also showed no significant improvement in the 5-year LRRFS, DFS, or OS of the PMRT group in a study of 151 ypN0 patients with clinical stage II-III disease (p > 0.05) [21]. Wang et al. conducted a meta-analysis of 12 studies and showed that PMRT was associated with reduced LRR instead of DFS or OS [22].

Given these conflicting results, this study aimed to investigate the role of PMRT in cT1-2N1 breast cancer patients who converted to ypN0 after NAC. The real-world study was designed to provide insight into the effect of PMRT without strict eligibility criteria. Therefore, it can better facilitate physicians’ comprehension and determination in practical applications. The rate of conversion from cN + to ypN0 after NAC in our study of 22.9% is consistent with what has been quoted in recent studies [7].

Despite the nonrandomized nature of our study, clinicopathological baseline variables showed no significant difference between the two groups, except for the systemic protocol (Table 1). Patients who received endocrine or targeted therapy were more likely to receive PMRT (p = 0.025, p < 0.001, respectively). Since this is a retrospective study, it seems that clinicians tended to recommend holistic systemic regimens, including PMRT for those patients they perceived to have more aggressive tumor features. The hypothesis that PMRT can improve outcomes in this cohort is actually influencing clinical treatment decisions. Patients who chose to omit PMRT were inclined to omit other systemic therapies as well, possibly due to economic factors and treatment preferences.

In the present cohort, PMRT did not provide any significant survival difference (Fig. 2). To exclude the interference of different systemic protocols, we also conducted disease-free survival analyses in HR( +) or HER2( +) cohorts with or without corresponding therapy (Fig. 3). In every subgroup, PMRT showed no significant DFS benefit (all p > 0.05). By univariate and multivariate analyses (Table 2 and Table 3), we confirmed that PMRT is not an independent predictor of DFS or OS (all p > 0.05). Moreover, after stratifying by recognized risk factors, we consistently found comparable OS and DFS in all subgroups (Table 4).

**Table 4:** Subgroup analysis of disease-free survival and overall survival for all included patients

Subgroup	DFS		OS	
	HR(95%CI)	p value	HR(95%CI)	p value
Age				
< 50	1.759(0.158–19.629)	0.426	0.035(0–403,799.201)	0.686
≥ 50	3.006(0.410–22.019)	0.103	2.657(0.165–42.694)	0.490
Hormonal receptor status				
Negative	324.557(0–8.602E11)	0.601	401.371(0–4.586E12)	0.612
Positive	1.494(0.271–8.232)	0.645	0.031(0–18,634.170)	0.608
HER-2 receptor status				
Negative	301.836(0–5.05E11)	0.598	–	–
Positive	1.900(0.334–10.816)	0.469	1.207(0.121–12.015)	0.873
Ki67				
≤ 30	2.129(0.345–13.131)	0.416	0.033(0–5915.501)	0.580
> 30	3.027(0.187–48.903)	0.435	359.345(0–1.877E12)	0.606
Clinical T stage				
T1	0.033(0–12049155.370)	0.734	0.037(0–462855.052)	0.693
T2	3.186(0.631–16.078)	0.161	2.510(0.157–40.127)	0.515
In-breast response				
Non-pCR	2.076(0.343–12.551)	0.426	1.285(0.125–13.181)	0.833
pCR	4.243(0.218–82.704)	0.340	–	–

*HER2* Human epidermal growth factor receptor 2, *pCR* Pathological complete response

Notably, all seven events of DFS were distant metastases rather than locoregional relapses. The complete response to NAC in nodal disease does represent a satisfying locoregional control.

In summary, despite the potential disadvantage of inadequate treatment, the no-PMRT group still showed a survival rate not inferior to that of PMRT group. This further indicates that the survival benefit of irradiation is not as significant in patients with favorable locoregional control and prognosis, such as our cohort. PMRT may need a more cautious assessment when systemic management options are recommended.

This study had several limitations. First, it is limited by its small sample size, retrospective nature, and nonrandomization. However, it provides insights into local Chinese patients’ situations for the first time. Second, not all initial nodal statuses were biopsy-proven. As a result, some patients with pathologically benign but radiographically concerning lymphadenopathy may be inappropriately included. There is a potential influence on the survival results. Last but not least, we only focused on the need for PMRT following neoadjuvant chemotherapy. Now that nonchemo neoadjuvant therapy is approved in the neoadjuvant setting, caution must be applied before extending our findings to another neoadjuvant intervention. On all accounts, our results support the need for future prospective, randomized studies to evaluate the impact of PMRT for cT1-2N1 patients who achieve nodal pCR after NAC.

## Conclusion

Our study showed that PMRT was not associated with OS or DFS benefits in cT1-2N1 breast cancer patients who converted to ypN0 after NAC and total mastectomy. Based on these data, it is difficult to make a definitive treatment recommendation. Our study still provides additional evidence for the safe omission of PMRT. More prospective research is required to establish a greater degree of accuracy on the impact of PMRT.

## Data Availability

The data used and analyzed during the current study are available from the corresponding author on reasonable request.

## Abbreviations

PMRT: Postmastectomy radiation therapy
NAC: Neoadjuvant chemotherapy
ypN0: Negative axillary nodes after neoadjuvant chemotherapy
pCR: Pathological complete response
TNM: Tumor-node-metastasis
ER: Estrogen receptor
PR: Progesterone receptor
HR: Hormone receptor
HER2: Human epidermal growth factor receptor 2
DFS: Disease-free survival
OS: Overall survival
LRR: Local–regional recurrence
DMFS: Distant metastasis-free survival
HRs: Hazard ratios
CIs: Confidence intervals
